# 2,2′-Dichloro-1,1′-[(pentane-1,5-diyldi­oxy)bis­(nitrilo­methyl­idyne)]dibenzene

**DOI:** 10.1107/S1600536809027433

**Published:** 2009-07-18

**Authors:** Wen-Kui Dong, Jun-Feng Tong, Jian-Chao Wu, Li Li, Jian Yao

**Affiliations:** a School of Chemical and Biological Engineering, Lanzhou Jiaotong University, Lanzhou 730070, People’s Republic of China

## Abstract

The mol­ecule of the title compound, C_19_H_20_Cl_2_N_2_O_2_, which lies across a crystallographic inversion centre, adopts a linear configuration. The dihedral angle between the two halves of the mol­ecule is 5.14 (2)°. In the crystal structure, inter­molecular C—H⋯O hydrogen bonds link neighbouring mol­ecules into an infinite zigzag chain supra­molecular structure.

## Related literature

For background to Schiff base compounds in transition metal coordination chemistry, see: Granovski *et al.* (1993 ▶). For the properties of Schiff base–metal complexes, see: Ghosh *et al.* (2006 ▶); Ward (2007 ▶). For our work on the synthesis and structural characterization of Schiff base–bis­oxime compounds, see: Dong *et al.* (2008*a* ▶). For related structures, see: Dong *et al.* (2008*b* ▶, 2009 ▶); Sun *et al.* (2009 ▶).
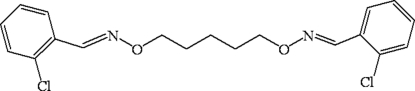


## Experimental

### 

#### Crystal data

C_19_H_20_Cl_2_N_2_O_2_
*M* *_r_* = 379.27
Monoclinic, 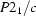

*a* = 12.5025 (12) Å
*b* = 19.7801 (17) Å
*c* = 7.8085 (9) Å
β = 96.747 (1)°
*V* = 1917.7 (3) Å^3^
*Z* = 4
Mo *K*α radiation
μ = 0.35 mm^−1^
*T* = 298 K
0.45 × 0.30 × 0.28 mm

#### Data collection

Bruker SMART CCD area-detector diffractometer
Absorption correction: multi-scan (*SADABS*; Sheldrick, 1996 ▶) *T*_min_ = 0.857, *T*_max_ = 0.908
9529 measured reflections
3376 independent reflections
1631 reflections with *I* > 2σ(*I*)
*R*_int_ = 0.048

#### Refinement

*R*[*F* ^2^ > 2σ(*F* ^2^)] = 0.045
*wR*(*F* ^2^) = 0.116
*S* = 1.02
3376 reflections
226 parameters
H-atom parameters constrained
Δρ_max_ = 0.21 e Å^−3^
Δρ_min_ = −0.25 e Å^−3^

### 

Data collection: *SMART* (Siemens, 1996 ▶); cell refinement: *SAINT* (Siemens, 1996 ▶); data reduction: *SAINT*; program(s) used to solve structure: *SHELXS97* (Sheldrick, 2008 ▶); program(s) used to refine structure: *SHELXL97* (Sheldrick, 2008 ▶); molecular graphics: *SHELXTL* (Sheldrick, 2008 ▶); software used to prepare material for publication: *SHELXTL*.

## Supplementary Material

Crystal structure: contains datablocks global, I. DOI: 10.1107/S1600536809027433/hg2535sup1.cif

Structure factors: contains datablocks I. DOI: 10.1107/S1600536809027433/hg2535Isup2.hkl

Additional supplementary materials:  crystallographic information; 3D view; checkCIF report

## Figures and Tables

**Table 1:** Hydrogen-bond geometry (Å, °)

*D*—H⋯*A*	*D*—H	H⋯*A*	*D*⋯*A*	*D*—H⋯*A*
C10—H10⋯O2^i^	0.93	2.60	3.527 (4)	177

Symmetry code: (i) 
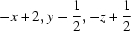
.

**Fig. 1.**
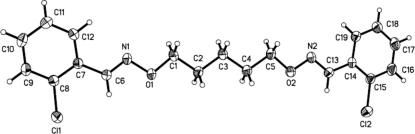
The molecule structure of the title compound with the atom numbering scheme. Displacement ellipsoids for non-hydrogen atoms are drawn at the 30% probability level.

**Fig. 2.**
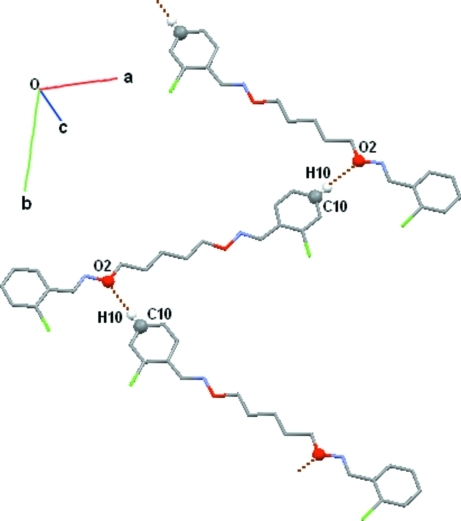
Part of zigzag chain supramolecular structure is formed by C—H···O intermolecular interactions with H bonds drawn as dashed lines. H atoms not involved in hydrogen bonding have been omitted for clarity.

## supplementary crystallographic information

### Comment

Schiff base compounds are one kind of important stereochemical models in transition metal coordination chemistry due to their ease of preparation and structural variations (Granovski *et al.*, 1993) and play an important role in the development of coordination chemistry owing to forming stable complexes with most of the transition metals or nontransition metals, in which many could exhibit intresting properties, including magnetic, optics and catalysis (Ghosh *et al.*, 2006; Ward *et al.*, 2007). In view of these facts and in continuation of our works on the synthesis and structural characterization of Schiff base bisoxime compounds (Dong *et al.*, 2008*a*), here we report synthesis and crystal structure of the title compound (Fig. 1).

The single-crystal structure of the title compound has a crystallographic inversion centre (symmetry code: -*x*, -*y*, -*z*) and twofold screw axis (symmetry code: -*x*, 1/2 + *y*, 1/2 - *z*), and adopts a linear configuration. This structure is not similar to what was observed in our previously reported series bisoxime compounds containing five-methene bridge, which assume a W-shape configuration (Dong *et al.*, 2008*b*) and distorted *Z* configuration (Sun *et al.*, 2009). The dihedral angle between the two halves of the molecule is 5.14 (2)°. Intermolecular C—H···O hydrogen bonds (Table 1, Fig. 2) link the neighbouring molecules into an infinite zigzag chain supramolecular structure.

### Experimental

2,2'-Dichloro-1,1'-[(pentane-1,5-diyldioxy)bis(nitrilomethylidyne)]dibenzene was synthesized according to an analogous method reported earlier (Dong *et al.*, 2009). To an ethanol solution (4 ml) of *o*-chlorobenzaldehyde (394.1 mg, 2.80 mmol) was added an ethanol absolute (3 ml) of 1, 5-bis(aminooxy)pentane (187.9 mg, 1.40 mmol). The mixture solution was stirred at 328 K for 8 h. After cooling to room temperature, no precipitate was formed, when the mixture solution was concentrated to about 1 ml under reduced pressure, and cooled to room temperature, the precipitate was filtered, and washed successively with ethanol and n-hexane, respectively. The product was dried under vacuum and purified with recrystallization from ethanol to yield 119.1 mg of the title compound. Yield, 24.7%. m. p. 327–328 K. Anal. Calcd. for C_19_H_20_Cl_2_N_2_O_2_: C, 60.17; H, 5.32; N, 7.39. Found: C, 60.10; H, 5.53; N, 7.27.

Colorless needle-like single crystals suitable for X-ray diffraction studies were obtained after one month by slow evaporation from a methanol solution of the title compound.

### Refinement

Non-H atoms were refined anisotropically. H atoms were treated as riding atoms with distances C—H = 0.97 Å (CH_2_), 0.93 Å (CH), and *U*_iso_(H) = 1.20 *U*_eq_(C).

### Crystal data

**Table:**

C_19_H_20_Cl_2_N_2_O_2_	*F*(000) = 792
*M**_r_* = 379.27	*D*_x_ = 1.314 Mg m^−^^3^
Monoclinic, *P*2_1_/*c*	Melting point = 327–328 K
Hall symbol: -P 2ybc	Mo *K*α radiation, λ = 0.71073 Å
*a* = 12.5025 (12) Å	Cell parameters from 1696 reflections
*b* = 19.7801 (17) Å	θ = 2.6–21.6°
*c* = 7.8085 (9) Å	µ = 0.35 mm^−^^1^
β = 96.747 (1)°	*T* = 298 K
*V* = 1917.7 (3) Å^3^	Needle-like, colorless
*Z* = 4	0.45 × 0.30 × 0.28 mm

### Data collection

**Table:**

Bruker SMART CCD area-detector diffractometer	3376 independent reflections
Radiation source: fine-focus sealed tube	1631 reflections with *I* > 2σ(*I*)
graphite	*R*_int_ = 0.048
φ and ω scans	θ_max_ = 25.0°, θ_min_ = 1.6°
Absorption correction: multi-scan (*SADABS*; Sheldrick, 1996)	*h* = −11→14
*T*_min_ = 0.857, *T*_max_ = 0.908	*k* = −23→19
9529 measured reflections	*l* = −9→9

### Refinement

**Table:**

Refinement on *F*^2^	Primary atom site location: structure-invariant direct methods
Least-squares matrix: full	Secondary atom site location: difference Fourier map
*R*[*F*^2^ > 2σ(*F*^2^)] = 0.045	Hydrogen site location: inferred from neighbouring sites
*wR*(*F*^2^) = 0.116	H-atom parameters constrained
*S* = 1.02	*w* = 1/[σ^2^(*F*_o_^2^) + (0.0407*P*)^2^ + 0.181*P*] where *P* = (*F*_o_^2^ + 2*F*_c_^2^)/3
3376 reflections	(Δ/σ)_max_ = 0.001
226 parameters	Δρ_max_ = 0.21 e Å^−^^3^
0 restraints	Δρ_min_ = −0.25 e Å^−^^3^

### Special details

**Table:**

Geometry. All e.s.d.'s (except the e.s.d. in the dihedral angle between two l.s. planes) are estimated using the full covariance matrix. The cell e.s.d.'s are taken into account individually in the estimation of e.s.d.'s in distances, angles and torsion angles; correlations between e.s.d.'s in cell parameters are only used when they are defined by crystal symmetry. An approximate (isotropic) treatment of cell e.s.d.'s is used for estimating e.s.d.'s involving l.s. planes.
Refinement. Refinement of *F*^2^ against ALL reflections. The weighted *R*-factor *wR* and goodness of fit *S* are based on *F*^2^, conventional *R*-factors *R* are based on *F*, with *F* set to zero for negative *F*^2^. The threshold expression of *F*^2^ > σ(*F*^2^) is used only for calculating *R*-factors(gt) *etc*. and is not relevant to the choice of reflections for refinement. *R*-factors based on *F*^2^ are statistically about twice as large as those based on *F*, and *R*- factors based on ALL data will be even larger.

### Fractional atomic coordinates and isotropic or equivalent isotropic displacement parameters (Å^2^)

**Table:**

	*x*	*y*	*z*	*U*_iso_*/*U*_eq_	
Cl1	1.35447 (6)	0.01799 (4)	0.52460 (14)	0.0757 (4)	
Cl2	0.26416 (7)	0.37373 (5)	−0.36888 (13)	0.0750 (3)	
N1	1.03773 (19)	−0.03387 (13)	0.2814 (3)	0.0518 (7)	
N2	0.38680 (19)	0.17370 (14)	−0.2572 (3)	0.0548 (8)	
O1	0.98008 (15)	0.02573 (10)	0.2391 (3)	0.0561 (6)	
O2	0.48710 (16)	0.18002 (11)	−0.1578 (3)	0.0676 (7)	
C1	0.8763 (2)	0.00878 (16)	0.1514 (4)	0.0547 (9)	
H1A	0.8361	−0.0179	0.2260	0.066*	
H1B	0.8844	−0.0175	0.0488	0.066*	
C2	0.8178 (2)	0.07350 (15)	0.1030 (4)	0.0500 (9)	
H2A	0.8567	0.0985	0.0231	0.060*	
H2B	0.8159	0.1010	0.2054	0.060*	
C3	0.7034 (2)	0.06076 (15)	0.0203 (4)	0.0508 (9)	
H3A	0.7054	0.0333	−0.0823	0.061*	
H3B	0.6646	0.0357	0.1000	0.061*	
C4	0.6438 (2)	0.12609 (15)	−0.0285 (4)	0.0514 (9)	
H4A	0.6416	0.1533	0.0744	0.062*	
H4B	0.6833	0.1513	−0.1071	0.062*	
C5	0.5302 (2)	0.11490 (16)	−0.1123 (4)	0.0572 (10)	
H5A	0.5302	0.0868	−0.2141	0.069*	
H5B	0.4876	0.0927	−0.0327	0.069*	
C6	1.1294 (2)	−0.02145 (15)	0.3602 (4)	0.0478 (9)	
H6	1.1501	0.0231	0.3829	0.057*	
C7	1.2036 (2)	−0.07616 (16)	0.4163 (4)	0.0427 (8)	
C8	1.3086 (2)	−0.06409 (15)	0.4923 (4)	0.0481 (8)	
C9	1.3784 (2)	−0.11648 (18)	0.5446 (4)	0.0592 (10)	
H9	1.4480	−0.1071	0.5947	0.071*	
C10	1.3447 (3)	−0.18221 (19)	0.5225 (5)	0.0696 (11)	
H10	1.3913	−0.2176	0.5573	0.084*	
C11	1.2412 (3)	−0.19537 (18)	0.4484 (5)	0.0693 (11)	
H11	1.2182	−0.2399	0.4323	0.083*	
C12	1.1719 (2)	−0.14340 (17)	0.3981 (4)	0.0551 (9)	
H12	1.1020	−0.1534	0.3506	0.066*	
C13	0.3452 (2)	0.23100 (17)	−0.2923 (4)	0.0554 (10)	
H13	0.3823	0.2697	−0.2528	0.066*	
C14	0.2399 (2)	0.23755 (16)	−0.3936 (4)	0.0434 (8)	
C15	0.1946 (2)	0.30034 (15)	−0.4349 (4)	0.0473 (8)	
C16	0.0941 (3)	0.30657 (19)	−0.5273 (4)	0.0618 (10)	
H16	0.0650	0.3491	−0.5532	0.074*	
C17	0.0375 (3)	0.2494 (2)	−0.5807 (5)	0.0651 (11)	
H17	−0.0305	0.2533	−0.6427	0.078*	
C18	0.0805 (3)	0.18646 (19)	−0.5430 (4)	0.0618 (10)	
H18	0.0420	0.1479	−0.5802	0.074*	
C19	0.1803 (2)	0.18066 (16)	−0.4505 (4)	0.0548 (9)	
H19	0.2088	0.1379	−0.4253	0.066*	

### Atomic displacement parameters (Å^2^)

**Table:**

	*U*^11^	*U*^22^	*U*^33^	*U*^12^	*U*^13^	*U*^23^
Cl1	0.0486 (5)	0.0534 (6)	0.1195 (9)	−0.0057 (4)	−0.0139 (5)	0.0063 (6)
Cl2	0.0702 (6)	0.0465 (6)	0.1062 (8)	0.0037 (4)	0.0024 (5)	0.0050 (5)
N1	0.0426 (17)	0.0486 (18)	0.061 (2)	0.0090 (13)	−0.0089 (14)	0.0017 (15)
N2	0.0388 (16)	0.0545 (19)	0.068 (2)	0.0067 (13)	−0.0065 (14)	0.0003 (16)
O1	0.0426 (13)	0.0461 (15)	0.0745 (17)	0.0089 (10)	−0.0153 (11)	−0.0005 (12)
O2	0.0467 (14)	0.0513 (15)	0.097 (2)	0.0068 (11)	−0.0232 (13)	0.0020 (14)
C1	0.0383 (18)	0.057 (2)	0.065 (2)	0.0020 (16)	−0.0098 (17)	0.0033 (19)
C2	0.048 (2)	0.046 (2)	0.054 (2)	0.0081 (15)	−0.0018 (16)	0.0015 (17)
C3	0.0424 (19)	0.050 (2)	0.056 (2)	0.0040 (15)	−0.0075 (16)	0.0031 (18)
C4	0.047 (2)	0.052 (2)	0.053 (2)	0.0066 (16)	−0.0069 (16)	0.0019 (18)
C5	0.047 (2)	0.051 (2)	0.070 (3)	0.0118 (16)	−0.0080 (18)	0.0019 (19)
C6	0.0413 (19)	0.041 (2)	0.059 (2)	0.0021 (16)	−0.0035 (17)	0.0016 (18)
C7	0.0390 (19)	0.044 (2)	0.045 (2)	0.0003 (15)	0.0024 (15)	0.0028 (16)
C8	0.0434 (19)	0.046 (2)	0.054 (2)	−0.0009 (16)	0.0019 (16)	0.0038 (17)
C9	0.041 (2)	0.058 (3)	0.076 (3)	0.0084 (17)	−0.0022 (18)	0.010 (2)
C10	0.063 (3)	0.054 (3)	0.088 (3)	0.0170 (19)	−0.005 (2)	0.014 (2)
C11	0.067 (3)	0.047 (2)	0.091 (3)	0.0034 (19)	−0.006 (2)	0.000 (2)
C12	0.043 (2)	0.051 (2)	0.068 (3)	−0.0020 (16)	−0.0066 (17)	0.0000 (19)
C13	0.049 (2)	0.041 (2)	0.074 (3)	0.0053 (16)	−0.0004 (19)	0.0019 (19)
C14	0.0370 (19)	0.049 (2)	0.044 (2)	0.0076 (16)	0.0047 (15)	0.0023 (17)
C15	0.048 (2)	0.044 (2)	0.051 (2)	0.0067 (16)	0.0080 (17)	0.0059 (17)
C16	0.053 (2)	0.058 (3)	0.073 (3)	0.0161 (19)	0.0045 (19)	0.015 (2)
C17	0.047 (2)	0.080 (3)	0.066 (3)	0.008 (2)	−0.0041 (19)	0.013 (2)
C18	0.051 (2)	0.066 (3)	0.067 (3)	0.0014 (18)	−0.0002 (19)	−0.005 (2)
C19	0.050 (2)	0.048 (2)	0.065 (3)	0.0079 (17)	−0.0004 (18)	0.0007 (19)

### Geometric parameters (Å, °)

**Table:**

Cl1—C8	1.731 (3)	C6—H6	0.9300
Cl2—C15	1.739 (3)	C7—C12	1.390 (4)
N1—C6	1.260 (3)	C7—C8	1.396 (4)
N1—O1	1.401 (3)	C8—C9	1.385 (4)
N2—C13	1.264 (3)	C9—C10	1.371 (4)
N2—O2	1.401 (3)	C9—H9	0.9300
O1—C1	1.434 (3)	C10—C11	1.379 (4)
O2—C5	1.425 (3)	C10—H10	0.9300
C1—C2	1.500 (4)	C11—C12	1.371 (4)
C1—H1A	0.9700	C11—H11	0.9300
C1—H1B	0.9700	C12—H12	0.9300
C2—C3	1.520 (4)	C13—C14	1.459 (4)
C2—H2A	0.9700	C13—H13	0.9300
C2—H2B	0.9700	C14—C15	1.387 (4)
C3—C4	1.518 (4)	C14—C19	1.393 (4)
C3—H3A	0.9700	C15—C16	1.378 (4)
C3—H3B	0.9700	C16—C17	1.373 (4)
C4—C5	1.508 (3)	C16—H16	0.9300
C4—H4A	0.9700	C17—C18	1.374 (4)
C4—H4B	0.9700	C17—H17	0.9300
C5—H5A	0.9700	C18—C19	1.371 (4)
C5—H5B	0.9700	C18—H18	0.9300
C6—C7	1.459 (4)	C19—H19	0.9300
			
C6—N1—O1	111.4 (2)	C12—C7—C6	121.1 (3)
C13—N2—O2	111.0 (3)	C8—C7—C6	122.2 (3)
N1—O1—C1	109.1 (2)	C9—C8—C7	121.7 (3)
N2—O2—C5	110.2 (2)	C9—C8—Cl1	118.2 (2)
O1—C1—C2	107.9 (2)	C7—C8—Cl1	120.1 (2)
O1—C1—H1A	110.1	C10—C9—C8	119.9 (3)
C2—C1—H1A	110.1	C10—C9—H9	120.0
O1—C1—H1B	110.1	C8—C9—H9	120.0
C2—C1—H1B	110.1	C9—C10—C11	119.4 (3)
H1A—C1—H1B	108.4	C9—C10—H10	120.3
C1—C2—C3	111.9 (2)	C11—C10—H10	120.3
C1—C2—H2A	109.2	C12—C11—C10	120.6 (3)
C3—C2—H2A	109.2	C12—C11—H11	119.7
C1—C2—H2B	109.2	C10—C11—H11	119.7
C3—C2—H2B	109.2	C11—C12—C7	121.7 (3)
H2A—C2—H2B	107.9	C11—C12—H12	119.2
C4—C3—C2	112.1 (2)	C7—C12—H12	119.2
C4—C3—H3A	109.2	N2—C13—C14	121.3 (3)
C2—C3—H3A	109.2	N2—C13—H13	119.4
C4—C3—H3B	109.2	C14—C13—H13	119.4
C2—C3—H3B	109.2	C15—C14—C19	117.4 (3)
H3A—C3—H3B	107.9	C15—C14—C13	121.5 (3)
C5—C4—C3	113.2 (2)	C19—C14—C13	121.0 (3)
C5—C4—H4A	108.9	C16—C15—C14	121.6 (3)
C3—C4—H4A	108.9	C16—C15—Cl2	118.3 (3)
C5—C4—H4B	108.9	C14—C15—Cl2	120.1 (2)
C3—C4—H4B	108.9	C17—C16—C15	119.4 (3)
H4A—C4—H4B	107.8	C17—C16—H16	120.3
O2—C5—C4	106.5 (2)	C15—C16—H16	120.3
O2—C5—H5A	110.4	C16—C17—C18	120.4 (3)
C4—C5—H5A	110.4	C16—C17—H17	119.8
O2—C5—H5B	110.4	C18—C17—H17	119.8
C4—C5—H5B	110.4	C19—C18—C17	119.8 (3)
H5A—C5—H5B	108.6	C19—C18—H18	120.1
N1—C6—C7	120.8 (3)	C17—C18—H18	120.1
N1—C6—H6	119.6	C18—C19—C14	121.3 (3)
C7—C6—H6	119.6	C18—C19—H19	119.3
C12—C7—C8	116.7 (3)	C14—C19—H19	119.3
			
C6—N1—O1—C1	−179.5 (3)	C9—C10—C11—C12	−0.5 (6)
C13—N2—O2—C5	177.0 (3)	C10—C11—C12—C7	1.3 (5)
N1—O1—C1—C2	−178.3 (2)	C8—C7—C12—C11	−1.5 (5)
O1—C1—C2—C3	−175.9 (2)	C6—C7—C12—C11	179.2 (3)
C1—C2—C3—C4	179.9 (3)	O2—N2—C13—C14	−179.5 (3)
C2—C3—C4—C5	179.5 (3)	N2—C13—C14—C15	−178.9 (3)
N2—O2—C5—C4	172.1 (2)	N2—C13—C14—C19	2.0 (5)
C3—C4—C5—O2	−177.2 (3)	C19—C14—C15—C16	0.6 (5)
O1—N1—C6—C7	−179.8 (3)	C13—C14—C15—C16	−178.5 (3)
N1—C6—C7—C12	−5.7 (5)	C19—C14—C15—Cl2	−179.9 (2)
N1—C6—C7—C8	175.1 (3)	C13—C14—C15—Cl2	1.0 (4)
C12—C7—C8—C9	0.9 (5)	C14—C15—C16—C17	−0.3 (5)
C6—C7—C8—C9	−179.8 (3)	Cl2—C15—C16—C17	−179.9 (3)
C12—C7—C8—Cl1	−178.7 (2)	C15—C16—C17—C18	−0.2 (5)
C6—C7—C8—Cl1	0.6 (4)	C16—C17—C18—C19	0.4 (5)
C7—C8—C9—C10	−0.1 (5)	C17—C18—C19—C14	−0.2 (5)
Cl1—C8—C9—C10	179.4 (3)	C15—C14—C19—C18	−0.4 (5)
C8—C9—C10—C11	−0.1 (5)	C13—C14—C19—C18	178.8 (3)

### Hydrogen-bond geometry (Å, °)

**Table:**

*D*—H···*A*	*D*—H	H···*A*	*D*···*A*	*D*—H···*A*
C10—H10···O2^i^	0.93	2.60	3.527 (4)	177

Symmetry codes: (i) −*x*+2, *y*−1/2, −*z*+1/2.
